# A System of Ophthalmic Operations

**Published:** 1911-07

**Authors:** 


### BOOKS AND AUTHORS

**A System of Ophthalmic Operations.** Being a Complete Treatise on the Operative Conduct of Ocular Diseases and Some Extraocular Conditions Causing Eye Symptoms. Edited and partly written by **Casey A. Wood**, M.D., C.M., D.C.L., Late Professor of Ophthalmology and Head of the Department, Northwestern University Medical School, Chicago. Complete in Two Volumes. Volumes I and II. Pp. 1824, with 968 illustrations. Chicago: Cleveland Press. 1911. (Cloth, $15.00).

In a work like this the reviewer's attention is naturally diected, first, to the personality of the chief editor. In this case, it is Casey A. Wood, who is well known throughout the ranks of English-speaking ophthalmologists for his scientific attainments, for his skill as a practitioner of ocular surgery and for his experience as an author and editorial writer of authority. His extensive association, also, with other ophthalmologists in educational and society work has eminently qualified him to select a staff consisting of thirty collaborators of experience, skill and reputation, and to assign to them the subjects in such a way that the whole list of surgical diseases and operations on the eye is covered. No question, therefore, can be raised as to the high character of the authorship of the work before us.

The editor very properly dedicated the work to Dr. Herman Knapp, "teacher, writer, counsellor," and he might have added, America's leader in ophthalmic surgery, of world wide fame, whose death following soon after its publication is lamented, not only in this country, but throughout Europe. For that reason the dedication now reverts to his memory and not to Dr. Knapp, the active surgeon.

The subjects of the work are divided into eight parts, the general plan being the same as that followed in the System of Ophthalmic Therapeutics, which was published recently and is intended to constitute volume one of a complete System of Ophthalmic Therapeutics and Operations, and the two volumes before us, as the editor says, "being a supplement to that treatise and to form with it a reasonable complete exposition of ocular therapy, both medical and surgical, from the earliest to the latest times."

The first chapter of the work, by M. Frank, cannot be said to have reference to surgical operations, but does refer to eminent ophthalmic surgeons of the past, whose history, in this connection, has a decided interest.

The editor has extended the subjects beyond those of ophthalmic operations and has wisely recognised that there should be included chapters treating on the surgery of organs distant from the eye and for the relief of such conditions as optic neuritis, with its attending symptoms, the etiology being mostly cerebral tumors, and the treatment, the recent procedures of brain decompression, this having been found to give marvelous abatement of the associated impairment of vision. The eminent surgeon, A. E. Halsted, writes this chapter. Another chapter includes operations, by Frank Brawley, on the nose and its accessory cavities, diseases of which often cause marked visual disturbances. Still another chapter has been introduced, which is a most opportune and valuable one and deals with the legal relations of ophthalmic surgery, by T. H. Shastid, and in view of the legal prosecutions to which every ophthalmic surgeon is constantly exposed, such a chapter has a proper place in a work like this.

The chapters comprising part III take up subjects of special importance to the beginner in ophthalmology, the first being the development of operative skill on the part of the surgeon, by A. E. Bulson, Jr., which is replete with helpful suggestions. The second chapter, by W. G. Byers and the succeeding ones, by E. C. Ellett, E. S. Thomson, W. Reber and T. A. Woodruff deal with the preoperative technic, after treatment, electric lighting and other electric appliances, both local and general anesthesia in ophthalmic operations, ophthalmic hospitals, wards, rooms and their furnishings, respectively. The chapters comprising part IV are written by such eminent ophthalmologists as Frank Allport, G. S. Derby, E. Jackson, H. V. Würdemann and C. A. Wood. The contributors to Part VI are W. T. Shoemaker, John Green, Jr., H. B. Chandler, C. A. Oliver, A. Alt, W. O. Nance,

W. C. Posey, C. A. Wood, D. W. Greene, P. A. Callan, M. Standish, C. H. Beard and W. H. Wilder. Parts VII and VIII conclude the work and are written by H. F. Hansell, J. H. Bell, F. E. Brawley and A. E. Halsted. The reputation of these authors gives assurance of the value of their articles.

The contributions of the chief editor should not be passed over without calling the attention of the reader to them, because of their unusual practical value. That on operations of the retina is of deep interest, giving a detailed historical review of the detachment of this structure and the operations and treatment best suited to its relief. Another article worthy of special reference, here, is that on extraction of the senile type of cataract. It is so complete in the considerations of it that, in fact, it constitutes a concise monograph on the subject, treating, as it does, of the history of cataract, of its early and modern methods of extraction, of the various knives and other instruments used, together with suggestions in regard to the preparation of the patient, of the after treatment, and the like. Dr. Wood also writes chapters on the minor surgery of the eye and operations on the orbit, which are full of important information.

Among various chapters of wide interest is that by D. W. Greene, on the intracapsular extraction of cataract. Impelled by the desire to make this operation practicable, Major Smith of the Jullundur Hospital, India, after experimenting on thousands of persons, devised a successful procedure. Dr. Greene describes the history of the method and the manner of its execution.

Such, briefly, is the outcome of the combined production of men noted for ophthalmic knowledge and skill. Furthermore, the text is generously and artistically illustrated wherever illustrations would add to clearness.

The editor and also the ophthalmologists of this country are to be congratulated on the completion of this monumental work. It has its imperfections and errors, but they are few and unimportant as compared to the thoroughness, care and scientific exactness maintained by most of the papers. This System should be in the possession of every English-speaking ophthalmic surgeon.

